# Genetic Variation in Vitamin B-12 Content of Bovine Milk and Its Association with SNP along the Bovine Genome

**DOI:** 10.1371/journal.pone.0062382

**Published:** 2013-04-23

**Authors:** Marc J. M. Rutten, Aniek C. Bouwman, R. Corinne Sprong, Johan A. M. van Arendonk, Marleen H. P. W. Visker

**Affiliations:** 1 Animal Breeding and Genomics Centre, Wageningen University, Wageningen, The Netherlands; 2 NIZO Food Research B.V., Ede, The Netherlands; University of California Davis, United States of America

## Abstract

Vitamin B-12 (also called cobalamin) is essential for human health and current intake levels of vitamin B-12 are considered to be too low. Natural enrichment of the vitamin B-12 content in milk, an important dietary source of vitamin B-12, may help to increase vitamin B-12 intake. Natural enrichment of the milk vitamin B-12 content could be achieved through genetic selection, provided there is genetic variation between cows with respect to the vitamin B-12 content in their milk. A substantial amount of genetic variation in vitamin B-12 content was detected among raw milk samples of 544 first-lactation Dutch Holstein Friesian cows. The presence of genetic variation between animals in vitamin B-12 content in milk indicates that the genotype of the cow affects the amount of vitamin B-12 that ends up in her milk and, consequently, that the average milk vitamin B-12 content of the cow population can be increased by genetic selection. A genome-wide association study revealed significant association between 68 SNP and vitamin B-12 content in raw milk of 487 first-lactation Dutch Holstein Friesian cows. This knowledge facilitates genetic selection for milk vitamin B-12 content. It also contributes to the understanding of the biological mechanism responsible for the observed genetic variation in vitamin B-12 content in milk. None of the 68 significantly associated SNP were in or near known candidate genes involved in transport of vitamin B-12 through the gastrointestinal tract, uptake by ileum epithelial cells, export from ileal cells, transport through the blood, uptake from the blood, intracellular processing, or reabsorption by the kidneys. Probably, associations relate to genes involved in alternative pathways of well-studied processes or to genes involved in less well-studied processes such as ruminal production of vitamin B-12 or secretion of vitamin B-12 by the mammary gland.

## Introduction

Vitamin B-12 (also called cobalamin) plays an important role in human health. Recently, Green [1] reviewed the health aspects of vitamin B-12. Macrocytic anemia and neurological disorder are well known symptoms of vitamin B-12 deficiency. In addition, vitamin B-12 may play a role in prevention of clinical entities as osteoporosis, neurocognitive decline, cardiovascular disease and neural tube defects in newborns.

Optimal vitamin B-12 intake may exceed the current recommended daily intake, which is 0.9–2.4 µg/d across lifespan in Canada and the USA [2]. Based on blood plasma levels, optimal vitamin B-12 intake may be as high as 4 to 10 µg/d [2], [3], [4]. Such a high intake of vitamin B-12 is not always achieved [5].

Dietary vitamin B-12 is found only in animal products, and dairy is an important source of vitamin B-12 in countries with a high dairy consumption. For example, in the Netherlands dairy provides approximately 40% of daily vitamin B-12 intake. In addition, observational studies suggest that vitamin B-12 from dairy products is better bioavailable than vitamin B-12 from other sources [6], [7]. Thus, natural enrichment of the vitamin B-12 content in milk may be a good way to increase dietary vitamin B-12 intake.

There might be potential for natural enrichment of the vitamin B-12 content in milk by increasing cobalt levels of dairy feed, because vitamin B-12 is synthesized by ruminal microbiota that use cobalt from the cow’s feed as precursor [8]. This potential seems limited, as it has been shown that milk vitamin B-12 content leveled off between 4.2 and 4.5 µg/L, despite significantly higher cobalt levels of the cow’s feed (0.93 mg/kg dry matter vs 0.57 mg/kg dry matter) [9].

Yet unknown is the potential for natural enrichment of the vitamin B-12 content in milk through genetic selection. Although vitamin B-12 is not directly synthesized by the cow herself, it cannot be ruled out that there is genetic variation between cows with respect to their milk vitamin B-12 content. Such genetic variation would enable genetic selection to increase the vitamin B-12 content in milk.

In the current study we quantified the genetic variation in vitamin B-12 content in milk present among Dutch Holstein Friesian cows. Furthermore, regions of the bovine genome associated with milk vitamin B-12 content were identified in a genome wide association study (GWAS).

## Results and Discussion

### Genetic Variation

**Figure 1:**
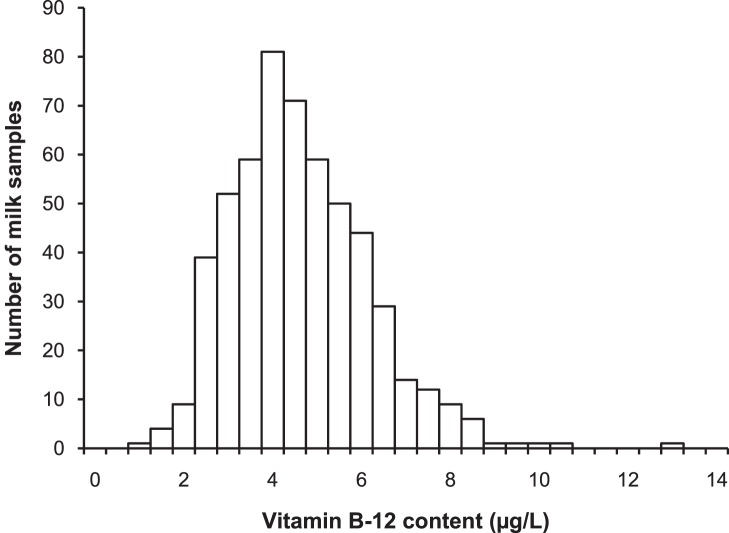
Vitamin B-12 content in 544 bovine milk samples. Frequency distribution of vitamin B-12 content in raw milk of 544 first-lactation Dutch Holstein Friesian cows.

Vitamin B-12 content varied from 1.0 to 12.9 µg/L, with a mean of 4.40±1.54 µg/L, in raw milk samples of 544 first-lactation Dutch Holstein Friesian cows. Figure 1 illustrates the large variation in milk vitamin B-12 content: the lowest 25% of samples had an average vitamin B-12 content of 2.62 µg/L and the highest 25% of samples had an average content of 6.45 µg/L, a difference of 3.83 µg/L. The mean content of vitamin B-12 in milk falls within the normal range reported in literature e.g. [10], [11].

Substantial amounts of farm variance and genetic variance in vitamin B-12 content in milk were detected among the 544 milk samples. Allocation to variance components for farm effects (0.40 µg/L^2^) and genetic effects (0.74 µg/L^2^) of the raw variance in milk vitamin B-12 content (2.42 µg/L^2^) is presented in Table 1. Variance components were allocated with a linear (animal) model that accounted for all known genetic relationships between animals [12]. Variation between farms as well as genetic variation between animals indicates that there is ample opportunity to influence the vitamin B-12 content in milk.

**Table 1:** Variance components and heritability of vitamin B-12 content in raw milk of 544 first-lactation Dutch Holstein Friesian cows.

Source of Variation	Vitamin B-12 (µg/L)
Farm ( 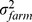 )	0.40±0.11
Genetic ( 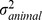 )	0.74±0.39
Residual ( 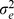 )	1.28±0.33
Phenotypic ( 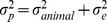 )	2.02±0.16
Heritability ( 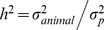 )	0.37±0.18

Variation between farms in vitamin B-12 content in milk may reflect differences between farms in feeding strategies, because cobalt from the cow’s feed is used by ruminal microbiota for vitamin B-12 synthesis [8]. Cobalt content can vary largely between different sources of roughage, mainly due to differences in soil [13]. Furthermore, roughage restriction has a lowering effect on milk vitamin B-12 content, especially in later lactation [14].

The amount of genetic variation between animals in vitamin B-12 content in milk is reflected by an estimated heritability of 0.37. This heritability, i.e. the proportion of variation due to genetic variation, is in the same range as heritabilities of e.g. test-day milk yield (0.41) and test-day fat yield (0.39) estimated in the same population [15]. Genetic variation between animals for milk vitamin B-12 content is, to our knowledge, demonstrated for the first time in the current study.

Genetic variation between animals in vitamin B-12 content in milk indicates that the genotype of the cow affects the amount of vitamin B-12 that ends up in her milk. This may suggest that the genotype of the cow influences the microbial processes in her rumen, because vitamin B-12 is synthesized by microorganisms in the bovine rumen [8]. However, variation in other processes such as vitamin B-12 absorbance from the digestive tract, transport through and uptake by the body, and secretion by the mammary gland may also contribute to genetic variation in milk vitamin B-12 content.

The fairly high heritability of 0.37 combined with a coefficient of variation of 35% for vitamin B-12 content in milk indicates that the average milk vitamin B-12 content of the cow population can be increased by genetic selection. Present-day genetic selection by dairy breeding organizations involves estimation of breeding values which requires phenotypes of literally many thousands of animals. Phenotypes for vitamin B-12 content in milk, as determined in the current study, are too expensive for such large-scale collection. Less expensive alternative phenotypes that correlate with milk vitamin B-12 content are not currently available. However, phenotype-based selection can also be substituted by genotype-based selection. Genotype-based selection requires knowledge on regions of the bovine genome associated with vitamin B-12 content in milk. In the next section, therefore, we report on a genome-wide association study to identify regions of the bovine genome associated with milk vitamin B-12 content. Identification of associated genomic regions also contributes to the understanding of the biological mechanism responsible for the observed genetic variation in vitamin B-12 content in milk.

### Genome-wide Association

**Figure 2:**
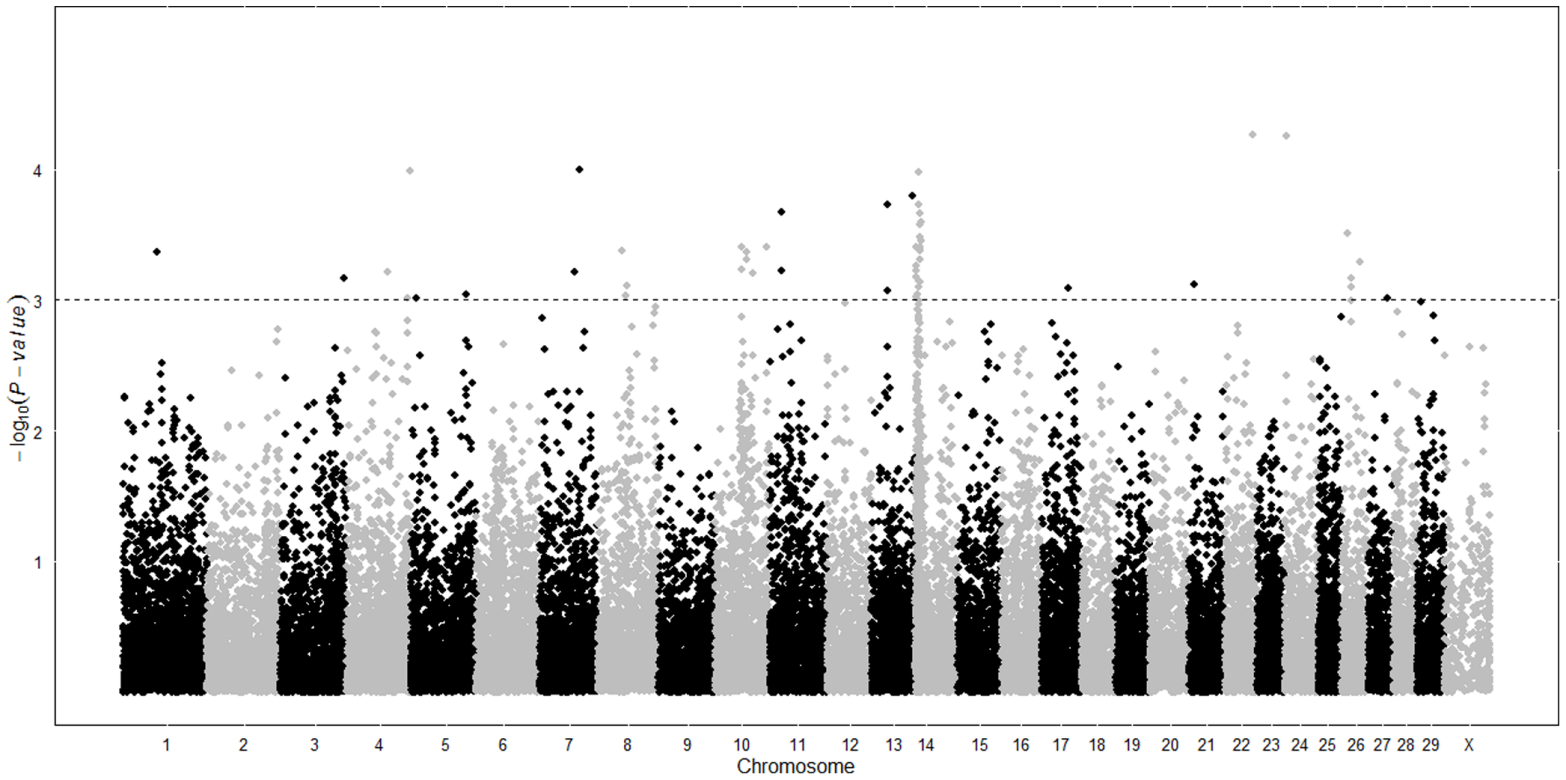
Association between 49,994 SNP and milk vitamin B-12 content of 487 cows. Significance (−log_10_(*P*-value)) of associations between 49,994 SNP along the bovine genome and vitamin B-12 content in raw milk of 487 first-lactation Dutch Holstein Friesian cows.

Significant association (−log_10_(*P*-value) >3) was found between 68 SNP and vitamin B-12 content in raw milk of 487 first-lactation Dutch Holstein Friesian cows (Table S1). Significantly associated SNP were spread over 16 *Bos taurus* (BTA) chromosomes. Figure 2 shows the distribution over the bovine genome of the significantly associated SNP among all 49,994 SNP that were tested for association with milk vitamin B-12 content. Among the 68 significantly associated SNP, clusters of at least 3 significantly associated SNP (within 10 Mbp) could be discriminated on BTA5, BTA8, BTA10, BTA13, BTA14 and BTA26. On BTA14 the number of significantly associated SNP might have been biased upward because the 50 K SNP chip covered this part of the genome more densely due to the proximity of the DGAT1 gene. SNP in or near DGAT1 (445 Kbp) were not significantly associated with vitamin B-12 content in milk. Additive effects were significant (−log_10_(*P*-value) >3) for most SNP and ranged between −0.51 and 0.81 µg/L milk vitamin B-12 (Table S1). Individual SNP explained between 2.2 and 4.0% of the phenotypic variance in milk vitamin B-12 content (Table S1) whereas together the significantly associated SNP explained 32% of the phenotypic variance. This is the first study showing associations between bovine SNP and milk vitamin B-12 content, thus, associations should be confirmed by an independent study to enhance their reliability.

None of the 68 significantly associated SNP were in or near genes that are known from human studies to be involved in transport of vitamin B-12 through the gastrointestinal tract (TCN1, GIF), uptake by ileum epithelial cells (CUBN, AMN), export from ileal cells (ABCC1/MRP1), transport through the blood (TCN1, TCN2), uptake from the blood (CD320), intracellular processing (LMBRD1, MMACHC, MMADHC, MMAB, MUT, MMAA, MTR, MTRR), or reabsorption by the kidneys (LRP2) [16], [17], [18]. This was established by examining the genes that were up to a distance of 100 Kbp of the significantly associated SNP (Table S1). SNP at further distance are unlikely to be in linkage disequilibrium (LD) with (un-typed) variation in the candidate genes, because useful LD (r^2^ = 0.20) extends to about 70 Kbp in the Dutch Holstein Friesian population [19].

Table 2 shows that most of the candidate genes for vitamin B-12 were actually genotyped for one or more SNP as part of the genome wide association study and that, indeed, none of these SNP were significantly associated with vitamin B-12 content in milk. However, the cluster of significantly associated SNP on BTA13 may have accounted for variation in milk vitamin B-12 content due to LD with variation in the *CUBN* gene, as some LD remains over longer distances. Three SNP in this significantly associated cluster at a distance of 118–121 Kbp from *CUBN* had significant test statistics (−log_10_(*P*-value) = 3.1), while two SNP in *CUBN* had test statistics that were close to significance (−log_10_(*P*-value) = 2.7). *CUBN* encodes cubilin, which is one of the two proteins that compose the cubam receptor. Cubam mediates the absorption of vitamin B-12 by the ileum epithelial cells where cubilin is required for recognition and binding the intrinsic factor-vitamin B-12 complex [17], [18], [20]. The significant associations on BTA13 suggest that some of the genetic variation in vitamin B-12 content in milk is due to variation in the uptake of vitamin B-12 from the gastrointestinal tract through variation in cubilin.

**Table 2:** Candidate genes for vitamin B-12 content in bovine milk with their genomic positions and significance of genotyped SNP.

Gene symbol	Gene name	Chromosome	Genomic position (Kbp)^a^	# SNP in gene^b^	Highest −log10(*P*-value)^b^
*LRP2*	low density lipoprotein receptor-relatedprotein 2 (megalin)	2	27,603,435.27,857,248	3	0.747
*MMADHC*	methylmalonic aciduria (cobalamindeficiency) cblD type	2	47,991,127.48,006,635	1	1.481
*MMACHC*	methylmalonic aciduria (cobalamindeficiency) cblC type	3	106,516,664.106,521,668	0	–
*CD320*	CD320	7	15,416,307.15,421,023	0	–
*LMBRD1*	LMBR1 domain containing 1	9	8,373,879.8,506,806	4	0.614
*CUBN*	Cubilin	13	31,053,631.31,312,338	10	2.699
*GIF*	gastric intrinsic factor	15	83,763,128.83,782,200	0	–
*TCN1*	transcobalamin I (haptocorrin)	15	83,791,407.83,806,715	1	1.319
*MMAA*	methylmalonic aciduria (cobalamindeficiency) cblA type	17	13,590,935.13,603,835	1	0.169
*MMAB*	methylmalonic aciduria (cobalamindeficiency) cblB type	17	66,719,631.66,730,086	0	–
*TCN2*	transcobalamin II	17	72,841,020.72,857,003	0	–
*MTRR*	5-methyltetrahydrofolate-homocysteinemethyltransferase reductase	20	68,917,318.68,946,683	1	1.051
*AMN*	Amnionless	21	67,751,621.67,762,225	0	–
*MUT*	methylmalonyl CoA mutase	23	22,488,908.22,530,197	1	0.206
*ABCC1*	ATP-binding cassette, sub-family C(CFTR/MRP), member 1	25	15,453,547.15,606,736	2	0.656
*MTR*	5-methyltetrahydrofolate-homocysteinemethyltransferase	28	7,953,588.8,080,514	2	0.599

a Based on genome assembly BTAU 4.6.1.

b Number of genotyped SNP in the gene and −log_10_(*P*-value) of the SNP that was most significantly associated with milk vitamin B-12 content.

The lack of association between SNP and most known candidate genes for vitamin B-12 indicates that variation in other genes causes most of the observed genetic variation in vitamin B-12 content in milk. Such other genes may be genes involved in alternative pathways of well-studied processes, as suggested to exist e.g. for export of vitamin B-12 from the cell [16], [18]. Other genes may also be involved in less well-studied processes such as ruminal production of vitamin B-12 or secretion of vitamin B-12 by the mammary gland. Regarding ruminal production of vitamin B-12; an effect of host genotype on the composition of its gut microbiota has been demonstrated in humans [21], [22]. In line with these results, it can be hypothesized that the genotype of the cow influences the composition of the microbiota in her rumen, which could have an impact on the ruminal vitamin B-12 synthesis and, consequently, on the availability of vitamin B-12 to the cow. Regarding secretion of vitamin B-12 by the mammary gland; uptake of vitamin B-12 from the blood by mammary epithelial cells is suggested to be similar as uptake by other body cells [23], mediated by CD320 [18]. Secretion of vitamin B-12 by mammary epithelial cells into milk might be similar as export from other body cells, thus, mediated by ABCC1 [18]. No SNP significantly associated with either CD320 or ABCC1 were found in the current study, indicating that possible variation in secretion of vitamin B-12 by the mammary gland is not due to variation in these genes.

### Conclusion

Genetic variation between animals in vitamin B-12 content in milk was established. Presence of genetic variation indicates that milk vitamin B-12 content can be increased by genetic selection. Genomic regions associated with vitamin B-12 content in milk were identified. These genomic regions can be targeted by marker assisted genetic selection that aims to increase milk vitamin B-12 content. Identification of genomic regions also shows that most known candidate genes for vitamin B-12 are not likely to be responsible for the observed genetic variation in vitamin B-12 content in milk.

## Materials and Methods

### Phenotypes

Vitamin B-12 content was determined in samples of raw morning milk taken in winter from 544 first-lactation Dutch Holstein Friesian cows. These 544 samples were a subset of the winter milk samples that were taken from 2,000 cows for the Dutch Milk Genomics Initiative. The 544 cows were housed on 148 farms throughout the Netherlands and on average nearly 4 cows per farm were sampled. The 544 cows were between 76 and 282 days in milk at the time of collecting the milk samples.

Vitamin B-12 content in the milk samples (µg/L) was determined as described by Winkels et al. [24]. Briefly, milk samples were freeze-dried and vitamin B-12 was extracted from the freeze-dried milk by heating at 119°C for 15 min with the use of 0.1 mol acetate buffer (pH 4.6) with 50 mg/L potassium cyanide. Vitamin B-12 was analyzed in the filtrated extract with a competitive binding radioassay with (^57^Co) cyanobalamin (SOP/VIT065; TNO Quality of life, Zeist, the Netherlands). The 544 milk samples were analyzed in 28 runs. In every run a control sample of skim milk powder was analyzed in duplicate in order to obtain the within-run variation and the between-run variation of the analyses. The within-run CV of milk vitamin B-12 content ranged from 0.1–7.1% (mean 2.3%), whereas the between-run CV was 3.0%.

### Quantitative Genetic Analysis

Variation in vitamin B-12 content in the milk samples of the 544 cows was quantified using the following animal model, adopted from Stoop et al. [25]:
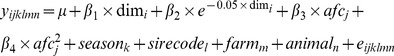
(1)where *y_ijklmn_* is an observation of milk vitamin B-12 content of animal *n* on farm *m*, with sirecode *l*, season of calving (season) *k*, age at first calving (afc) *j* and days in milk (dim) *i*; *µ* is the general mean; dim*_i_* is a covariate for the effect of days in milk, modeled with a Wilmink curve [26]; afc*_j_* is a covariate for the effect of age at first calving; season*_k_* is a fixed effect with 3 classes for season of calving, summer (June to August 2004), autumn (September to November 2004), and winter (December 2004 to February 2005); sirecode*_l_* is a fixed effect accounting for possible differences in genetic level between the groups of proven bull daughters and young bull daughters; farm*_m_* is a random effect for farm, distributed as N(0, I 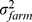) with identity matrix I and farm variance 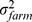; animal*_n_* is a random additive genetic effect for animal, distributed as N(0, A 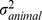), with additive genetic relationship matrix A based on a pedigree of 26,300 animals and additive genetic variance 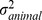; and e*_ijklmn_* is a random residual effect, distributed as N(0, I 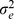) with identity matrix I and residual variance 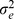. Model parameters were estimated by residual maximum likelihood (reml) implemented in ASReml software release 2.0 [27]. The effect of days in milk was nearly significant (*P*-value = 0.054), whereas the effects of age at first calving, season of calving and sirecode were not. The proportion of variation due to genetic variation, i.e. the intraherd heritability [28], was calculated as: 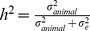. Random farm variance (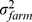) was excluded from the phenotypic variance in order to make the heritability comparable to other studies that model farm variance as fixed effect, as a result of which farm variance does not contribute to the phenotypic variance.

### Genotypes

Genomic DNA of the cows was genotyped for 50 K SNP markers. Genomic DNA was isolated with Puregene (Gentra, Qiagen) from whole blood samples of the cows. Blood samples were collected in accordance with the guidelines for the care and use of animals as approved by the ethical committee on animal experiments of Wageningen University (protocol: 200523.b). The DNA was genotyped with a 50 K Infinium SNP genotyping chip [29], designed by CRV (cooperative cattle improvement organization, Arnhem, the Netherlands) and obtained from Illumina (San Diego, CA, USA). The 50,855 technically successful SNP were assumed to map according to the bovine genome assembly BTAU 4.0 [30]. The average distance between SNP was 52,452 bp. The 776 SNP that did not map to any of the *Bos taurus* (BTA) chromosomes were assigned to BTA0 and remained part of the marker set. Analysis of unreliable or uninformative markers was prevented by discarding SNP with a genotyping rate <80% (n = 392, considering all genotyped cows of the Dutch Milk Genomics Initiative) and monomorphic SNP (n = 469, considering genotyped cows with milk vitamin B-12 phenotypes only) from the marker set. As a result, the final marker set comprised 49,994 SNP. The genome wide association study was done with data on 487 cows that had both SNP genotypes and phenotypes for milk vitamin B-12 content.

### Genome Wide Association Study

The bovine genome was screened for associations with vitamin B-12 content in milk by means of single SNP analyses. Model 1 was adjusted and extended for this purpose. First, the variance components estimated in the quantitative genetic analysis were fixed. Second, the SNP was added as a fixed effect. Solutions were generated individually for each of the 49,994 SNP using ASReml software release 2.0 [27]. Significantly associated SNP (−log_10_(*P*-value) >3) that had less than 10 observations for one of the genotype classes were removed from the results.

Additive and dominance effects of significantly associated SNP (−log_10_(*P*-value) >3) were estimated as contrasts between the relevant genotype classes, and tested for significance by adding both contrasts simultaneously to the adjusted animal model. The phenotypic variance explained by significantly associated SNP (−log_10_(*P*-value) >3) was calculated from the estimated genotype effects obtained from the adjusted and extended animal model and the observed genotype frequencies. The SNP variance is expressed as percentage of the phenotypic variance (
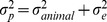
). These percentages can be overestimated, especially when the SNP effects are small, due to the so called Beavis effect [31]. To estimate the total phenotypic variance explained by the significantly associated SNP (−log_10_(*P*-value) >3) together, the adjusted animal model was extended with fixed effects for multiple SNP. Overestimation of the total variance explained is likely to result from adding all significantly associated SNP to the model because SNP at short distance may account for the same variance due to LD. Therefore, of multiple significantly associated SNP that were less than 10 Mbp apart, only the most significant SNP was added to the model. This resulted in an animal model that was extended with fixed effects for 23 SNP (indicated in Table S1). The total phenotypic variance explained was calculated from the estimated genotype effects obtained from the model that was extended with fixed effects for the 23 SNPs and the observed genotype frequencies of these SNPs.

### SNP in Candidate Genes

To establish whether known candidate genes for vitamin B-12 had been genotyped, sequences of the 50 K SNP markers were aligned with reference sequences of these candidate genes. Reference sequences of candidate genes were obtained from the NCBI database (http://www.ncbi.nlm.nih.gov/gene/) and alignment was performed using blast (http://blast.ncbi.nlm.nih.gov/).

### Outlier and Additional Data

The dataset on 544 cows only became this size after initial analyses led to removal of data on one cow and addition of data on 48 cows. The removal of data related to a milk sample with a vitamin B-12 content of 17.7 µg/L that influenced the results of both the quantitative genetic analysis and the genome wide association study considerably. This milk sample was designated as outlier after additional phenotyping of the same milk sample and two other samples of the same cow, and all data on this cow were discarded from the dataset. The addition of data related to significance of associations that could possibly be attributed to low-frequent genotypes. To exclude this possibility, 48 additional cows from the Dutch Milk Genomics Initiative that had the low-frequent genotypes were phenotyped for milk vitamin B-12 content and all their data were added to the dataset.

## Supporting Information

Table S1 **SNP significantly associated with vitamin B-12 content in milk of 487 cows.** All 68 SNP significantly associated with vitamin B-12 content in raw milk of 487 first-lactation Dutch Holstein Friesian cows, with for each SNP its −log_10_(*P*-value), genotype effects with standard errors (SE), minor allele frequency, additive and dominance SNP effects with SE and −log_10_(*P*-value)s, phenotypic variance explained, indication whether the SNP was added to the model to estimate the total phenotypic variance explained, and information on SNP position, sequence and proximity of genes.
(XLSX)
